# Tailoring photosensitive ROS for advanced photodynamic therapy

**DOI:** 10.1038/s12276-021-00599-7

**Published:** 2021-04-08

**Authors:** Duc Loc Sai, Jieun Lee, Duc Long Nguyen, Young-Pil Kim

**Affiliations:** 1 Department of Life Science, Hanyang University, Seoul, 04763 Republic of Korea; 2 Department of HY-KIST Bio-Convergence, Hanyang University, Seoul, 04763 Republic of Korea; 3 Research Institute for Natural Sciences, Hanyang University, Seoul, 04763 Republic of Korea; 4 Research Institute for Convergence of Basic Sciences, Hanyang University, Seoul, 04763 Republic of Korea; 5 Institute of Nano Science and Technology, Hanyang University, Seoul, 04763 Republic of Korea

**Keywords:** Biological therapy, Biosensors

## Abstract

Photodynamic therapy (PDT) has been considered a noninvasive and cost-effective modality for tumor treatment. However, the complexity of tumor microenvironments poses challenges to the implementation of traditional PDT. Here, we review recent advances in PDT to resolve the current problems. Major breakthroughs in PDTs are enabling significant progress in molecular medicine and are interconnected with innovative strategies based on smart bio/nanomaterials or therapeutic insights. We focus on newly developed PDT strategies designed by tailoring photosensitive reactive oxygen species generation, which include the use of proteinaceous photosensitizers, self-illumination, or oxygen-independent approaches. While these updated PDT platforms are expected to enable major advances in cancer treatment, addressing future challenges related to biosafety and target specificity is discussed throughout as a necessary goal to expand the usefulness of PDT.

## Photodynamic therapy: Bringing light into tumors to treat cancer

Advancements in photosensitive proteins, nanomaterials, and luminescence are improving the ability of photodynamic therapy (PDT) to attack cancerous tumors. In PDT photosensitive drugs are introduced into tumors, which are then exposed to light, producing reactive oxygen species that kill cells. Young-Pil Kim and coworkers at Hanyang University in Seoul, South Korea, reviewed the challenges of PDT, including drug side effects and how to deliver light into tumors. They highlight advances in protein-based photosensitive drugs, which avoid the side effects of their non-protein counterparts, and could even be generated within the body through genetic manipulation. Bioluminescent and chemiluminescent chemicals have been incorporated into nanomaterials such as quantum dots, carrying light deep into tumors. The use of hybrid oxygen-carrying proteins can provide oxygen for PDT, even inside oxygen-poor tumors that have depleted the local blood supply.

## Introduction

Since the first demonstration for bladder cancer treatment in 1976^1^, photodynamic therapy (PDT) has emerged as a clinically approved, noninvasive therapeutic regimen against a range of cancers and nonmalignant diseases^2–4^. PDT can not only suppress tumor growth, but can also stimulate an acute inflammatory response around locally treated tumors, thus encouraging antitumor immunity by releasing secondary inflammatory mediators^5–8^. Generally, PDT relies on three essential components: a photosensitizer (PS), oxygen, and light. The PS administered to the tumor site is activated by light of a specific wavelength, followed by the generation of cytotoxic reactive oxygen species (ROS) in the presence of oxygen; thus, the ROS generated by the PS is the key mechanism by which PDT leads to localized cell death and tissue devastation. In principle, a light-sensitized (excited) PS can react directly with a suitable substrate (unsaturated lipid, protein, or nucleic acid) to produce unstable radicals through proton or electron transfer (type I reaction), leading to oxygenated products in the presence of oxygen, such as a superoxide anion radical (O_2_^•−^), a hydroxyl radical (OH^•^), or hydrogen peroxide (H_2_O_2_). In turn, the excited PS can react with molecular oxygen to form singlet oxygen (^1^O_2_) through energy transfer (type II reaction); ^1^O_2_ is a major cytotoxin in PDT, especially at high oxygen contents^9,10^. Although the ratio between type I and type II reactions depends on the type and concentration of PS, the level of oxygen, and the irradiation degree, the mechanistic details between ROS generation and tumor ablation are not fully understood.

**Fig. 1:**
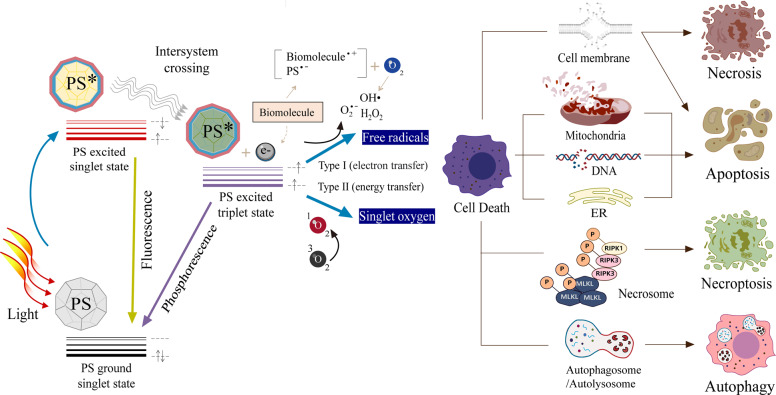
Schematic illustration of photodynamic reactions (either type I or type II) and cell death pathways in the process of PDT. A PS absorbs energy from light to kill tumor cells via ROS generation. The PDT-induced modes of cell death, including apoptosis, necrosis, necroptosis, and autophagy, depend on the cell type, PS type or concentration, intracellular localization, light dose, and oxygen partial pressure. PDT photodynamic therapy, PS photosensitizer, ER endoplasmic reticulum.

As illustrated in Fig. 1, ROS produced by PDT trigger cell death in different ways. The type of cell death induced by PDT depends on the cell type, the PS type or concentration, the intracellular localization, the light dose, and the oxygen partial pressure. Necrosis is a major cell death modality induced by PDT when a chemical PS is confined to the plasma membrane^11^. Apoptosis can also be induced by PDT through oxidative stress when the PS is located at the plasma membrane^12^, as well as in other cellular organelles, such as the nucleus^13^, mitochondria^14,15^, endoplasmic reticulum^16,17^, and lysosomes^18,19^. It was reported that PDT led to necroptosis through the formation of necrosomes containing receptor-interacting protein kinase 1 (RIP1) and RIP3^20,21^. Autophagy is another pathway to cell death by photosensitization, which begins with the formation of spherical autophagosomes that ultimately induce autolysosomes to break down cytoplasmic components^22,23^. The ROS-mediated effects of PDT on cell death have been reviewed in previous reports^24–26^.

Despite the significant progress in the use of PDT in cancer therapy, it still faces certain limitations, including the accumulation or photobleaching of photosensitizers, the limited delivery of light doses, and ineffectiveness in tumor hypoxia^27^. For example, tetrapyrrole drugs (e.g., porphyrin or chlorin) or relatively safe prodrugs (e.g., aminolevulinic acid) often cause sunlight-induced photodermatosis or hepatic porphyria^28,29^. Furthermore, light penetration was limited to 1−2 mm underneath the skin in a clinical study^30^, and the tumor hypoxia that mainly occurs in the central areas of most solid tumors was reported to be resistant to PDT^31^. To circumvent these issues, various PDT modalities have been recently developed in combination with smart materials or other therapeutic modalities.

Here, we review recent advances in PDTs to resolve the current problems. To avoid redundancy with general review articles on PDT, as reported previously^32–36^, we focus on innovative PDT strategies based on tailoring photosensitive ROS generation, which include the use of proteinaceous PS, self-illumination, or oxygen-independent methods. To expand the usefulness of PDT, we further discuss ROS generation and targeting models in advanced PDT and summarize recent research results.

### PDT with proteinaceous PS

The anticancer efficiency of PDT largely depends on the improvement of PSs. Much effort has been made to develop PSs as chemical drugs that preferentially accumulate in aberrant vasculature and tumor tissues, but not in normal tissues. The abilities of PSs have evolved through their first (oligomeric hematoporphyrin derivatives), second (pure and synthetic compounds), and third generations (nano- or polymer-conjugates of second-generation PSs with targeting moieties)^37^. These chemical drugs or prodrugs rely primarily on localized treatment via a passive targeting route (i.e., enhanced permeability retention (EPR) effects in leaky vasculature and tortuous blood vessels) and/or via low-density lipoprotein receptors overexpressed in tumor cells^38^. Nonetheless, the distribution and ROS generation of PS can vary in vivo, and the heterogeneous accumulation of PS in tumor regions makes it difficult to determine the light dose required to induce photodynamic effects and to avoid photobleaching reactions. Especially since chemical PSs capitalize on high ROS production at high concentrations, this method could lead to nonspecific side reactions or dark cytotoxicity. To increase water solubility and target specificity while decreasing toxicity, chemical PSs with high hydrophobicity and lipophilicity can be conjugated with protein carriers^39^ or antibodies^40^. However, this still requires an elaborate conjugation process and cannot prevent the side effects of released chemical PSs.

To resolve the innate problems of chemical PSs, a class of flavin-binding proteins (miniSOG, SOPP, and Pp2FbFP^L30M^) or fluorescence (FL) protein variants (KillerRed, SuperNova, mKate, and KillerOrange) have been identified and proven to have light-activated ROS-generating properties (Table 1). Among the ROS, two species (superoxide and singlet oxygen) are primarily generated from these proteins. Singlet oxygen is more reactive and damaging in biological systems because more cells lack antioxidant enzymes against singlet oxygen than against superoxide. Indeed, it is noteworthy that protein photoreceptors found in plants and marine invertebrates have been distinctly fine-tuned to adapt for high light-energy efficiency over their lifetime. As a result of this high energy yield, ROS can be produced in large quantities inside the cell, but organisms have evolved self-defense mechanisms against internal ROS production by precisely regulating multiple signaling pathways. Proteinaceous PSs are created by engineering photosensitive proteins derived from living organisms that evolved in various environments. A mini singlet-oxygen generator (miniSOG) is a green fluorescent protein variant (106 amino acids) engineered from the LOV2 domain of *Arabidopsis thaliana* phototropin 2^41^. The flavin mononucleotide (FMN) in miniSOG serves as a chromophore, as well as an endogenous cofactor^42^. MiniSOG/FMN, as a photosensitizing protein, was developed to yield higher ^1^O_2_ than free FMN. This protein was mutated at FMN-binding sites to further increase the ^1^O_2_ quantum yield, resulting in singlet-oxygen-producing protein (SOPP), SOPP2, and SOPP3^43,44^. Pp2FbFP^L30M^ is a site-directed variant (L30M) from a flavin-binding protein, Pp2FbFP, found in *Pseudomonas sputita*^45^. In contrast to its relatively low-FL quantum yield, it was reported that the generation of ^1^O_2_ in Pp2FbFP^L30M^ is approximately threefold higher than that of miniSOG^45^. KillerRed was the first genetically encoded red fluorescent protein with high ROS production^46^, whose generation of ROS was reported to be 1,000-fold higher than that of enhanced green fluorescent protein^46–48^. This 26-kDa-protein has a barrel shape with 11 antiparallel beta-sheets and a QYG (Glu^65^-Tyr^66^-Gly^67^) chromophore^48^, and predominantly undergoes type I reactions rather than type II reactions. However, KillerRed tends to form dimers^46^, which may prevent efficient fusion with other proteins of interest in applications. To overcome this limitation, SuperNova was identified in 2013 as a monomeric variant (mutations at six amino acids) of KillerRed^49^. Despite the monomeric PS, SuperNova showed a 5% increase in ^1^O_2_ generation and a 10% reduction in superoxide generation^49^. Monomeric KillerOrange (mKillerOrange)^50^ or SuperNova Green^51^ was further developed by mutating a single amino acid (Y66W) or double amino acids (V44A and Y66W) of SuperNova, enabling blue or green light illumination, respectively. An mKate variant called mKate2, a monomeric far-red fluorescent protein known to generate both superoxide and ^1^O_2_ was derived from TagRFP^52^.

**Table 1:** Photochemical properties of proteinaceous PSs.

Name	Ex/Em	MW (kDa)	Chromophore	ROS Type	*Q*_Y_	Ref.
miniSOG	448/500	14	FMN	^1^O_2_	0.47	^41^
SOPP	439/488(515)	14	FMN	^1^O_2_	0.25	^43,44^
Pp2FbFP^L30M^	449/495	30	FMN	^1^O_2_	0.25	^45^
KillerRed	585/610	27	Q^65^Y^66^G^67^	O_2_^•−^	0.25	^46–48^
SuperNova	579/610	29	Q^65^Y^66^G^67^	^1^O_2_/ O_2_^•−^	0.30	^49^
mKillerOrange	512/555	27	Q^65^W^66^G^6^	O_2_^•−^	0.42	^50^
SuperNova Green	440/510	29	A^44^Q^65^W^66^G^67^	O_2_^•−^	0.23	^51^
mKate2	588/633	26	M^65^Y^66^G^6^	^1^O_2_/ O_2_^•−^	0.4	^52^

*PS* photosensitizer, *Ex* excitation wavelength, *Em* emission wavelength, *MW* molecular weight, *ROS* reactive oxygen species, *QY* fluorescence quantum yield, *Ref.* references, *FMN* flavin mononucleotide, *SOG* single-oxygen generator, *SOPP* singlet-oxygen photosensitizing protein.

These photosensitizing proteins have remarkable advantages over chemical PSs. First, upon light illumination, chromophores of proteinaceous PSs are capable of a high rate of intersystem crossing to generate ROS, compared to chemical dyes^53^. Second, they can be designed as genetically encoded proteins that are locally expressed in subcellular organelles with appropriate signal sequences, enabling precisely targeted and spatially controlled photodynamic action at the intracellular level. Third, the photodynamic action of locally expressed proteins has little effect on molecules in other regions, due to the ability to control the spatial distribution and the reaction time to light. For example, superoxide anions are known to have a lifetime of <15 min and a diffusion distance of <0.5 μm^54^, whereas singlet oxygen has a shorter lifetime of <0.04 μs and a more limited effective diffusion distance of <20 nm at the site of PS localization in the cell^55,56^. Therefore, with the diminutive lifespan of ROS, it is reasonable that if proteinaceous PDT is precisely regulated at the site of expression, it can perform rapid local treatment without damage to nontarget molecules under a given light and time condition. It is also important to note that proteinaceous PSs have lower phototoxicities than chemical PSs in the dark^57^. Significantly, photodynamic cytotoxicity or tumor devastation has been achieved using either protein-encoding genes transfected into mammalian cells^58,59^ or bacterially expressed proteins^60^. By this principle, proteins bound with proteinaceous PS can functionally be knocked out in living cells, which is known as chromophore-assisted light inactivation, an optogenetic ablation method with high spatiotemporal resolution^61,62^. For the delivery of proteinaceous PS-encoding genes, a tumor-specific delivery system is required, and low-expression genes must be overcome, but this method is expected to facilitate the usability of PDT in basic and applied life science research.

### PDT with self-illumination

Despite recent advances in the field of PSs, the efficient delivery of light energy to PSs is still a challenge because external light sources are limited in tissue permeability and cannot reach tumors deep within the body or behind organs. Even near-infrared light, generated by improved laser technology, cannot easily travel deeper than a few millimeters into a tissue or organ^63^. Most PSs absorb strongly at approximately 400 nm and weakly at 600−800 nm^64^, which causes a serious drop in the efficiency of ROS generation in vivo. Alternatively, two external light-independent methods, bioluminescence (BL) and chemiluminescence (CL), have been attractive because efficient energy transfer is possible through the induction of internal light near cancer tissues without the need for a high-intensity pulsed light source or expensive light-generating devices^65^. The external light-independent applications in PDT are summarized in Table 2.

**Table 2:** Representative studies on self-illuminating PDTs.

Energy source (component)	Transducer or carrier	Photosensitizer (type)	Targeting strategy (target)	Ref.
CL (luminol/H_2_O_2_)	Oligo (*p*-phenylene vinylene)	Oligo (*p*-phenylene vinylene) (Chemical)	Passive targeting (xenograft mice of HeLa; Fungus, *C. albicans*)	^69^
CL (luminol/H_2_O_2_)	Polymer dot	m-THPC (Foscan) (Chemical)	Active targeting (C6 and MCF-7 cells)	^70^
CL (CPPO/H_2_O_2_)	Pluronic F-127	TPE-BT-DC (Chemical)	Passive targeting (4T1 cells and xenograft mouse)	^71^
CL (luminol/H_2_O_2_)	PEG polymer	Chlorine e6 (Chemical)	Passive targeting (macrophage, some cancer cells, and inflammation mouse)	^72^
CL (luminol/H_2_O_2_)	Carbon dot	Chlorine e6 (Chemical)	Passive targeting (SMMC-7721 cells and xenograft mouse)	^73^
BL (Fluc/luciferin)	Gene transfection agent	Rose bengal (Chemical)	Passive targeting (NIH 3T3 cells)	^79^
BL (*R*luc variant/CTZ)	Quantum dot	m-THPC (Foscan) (Chemical)	Passive targeting (A549 cells and xenograft mice)	^80^
BL (*R*luc variant/CTZ)	Quantum dot	Chlorine e6 (Chemical)	Passive targeting (B16F10, CT26, or LLC cells and their xenograft mice)	^81^
BL (Fluc/luciferin)	PLGA nanoparticle	Rose bengal (Chemical)	Passive targeting (MCF-7 and HeLa cells)	^82^
BL (*G*luc variant/CTZ)	Mini-ferritin	Protoporphyrin IX (Chemical)	Passive targeting (SK-BR-3 and MDA-MB-231 cells)	^83^
BL (Nluc)	Gene transfection agent	miniSOG (Protein)	Passive targeting (SK-BR-3 cells)	^84^
BL (*R*luc variant/CTZ)	None	KillerRed, miniSOG (Protein)	Active targeting (Six BC cells, patient primary cells, and BC-xenograft mouse)	^60^

*CL* chemiluminescence, *BL* bioluminescence, *m-THPC* meta-tetra(hydroxyphenyl)-chlorin, *CPPO* bis[2,4,5-trichloro-6-(pentyloxycarbonyl) phenyl] oxalate, *TPE-BT-DC* methoxy-substituted tetraphenylethylene (TPE), benzothiadiazole (BT), and dicyanovinyl (DC), *PLGA* poly(lactide-co-glycolide), *Fluc* firefly luciferase, *R*luc *Renilla* luciferase, *G*luc *Gaussia* luciferase, Nluc, NanoLuc® luciferase, CTZ coelenterazine, BC breast cancer.

CL is the light emitted from a chemical reaction primarily between a peroxide (O−O)-containing molecule and an electron-rich group. The oxidation of luminol is a representative CL reaction; it occurs in an alkaline solution in the presence of hydrogen peroxide and oxidant catalysts, such as Fe^2+^, Cu^2+^, Co^2+^, periodate ions, or hydrogen peroxidase^66^. The oxidation product from luminol emits a strong blue CL at a maximum of 425 nm^67^, and is widely used in immunoassays with horseradish peroxidase (HRP)-conjugated antibodies^68^. Owing to their simplicity and the effectiveness of their light generation and ^1^O_2_ production, CL-generating systems have been developed for PDT. An initial report on CL-based PDT was published in 2012 by Wang’s group^69^. They used luminol and hydrogen peroxide as CL-generating molecules and cationic oligo(*p*-phenylene vinylene) as a PS. CL without external light irradiation activated PS, killing the cancer cells or pathogenic fungus (*Candida albicans*) surrounding the complex through ROS generation. A smart material based on CL was described, which used semiconducting polymer dots (Pdots) with an incorporated chemical PS, meta-tetra(hydroxyphenyl)-chlorin (m-THPC), which was conjugated with HRP (a CL catalyst) and folic acid (a tumor-targeting ligand)^70^. The CL induced by luminol/H_2_O_2_ on the surfaces of Pdots showed increased cytotoxicity in C6 glioma cells and MCF-7 breast cancer cells that overexpressed folate receptors on their cell membranes. In particular, Pdots have a large Stokes shift at excitation/emission ranging from 420 nm to 650 nm, leading to highly efficient ROS production via simultaneous CL resonance energy transfer (CRET, between luminol and Pdots) and FL resonance energy transfer (FRET, between Pdots and m-THPC). Similar to Pdots, organic nanoparticles (NPs) were synthesized by coupling electron donors and acceptors inside the NPs to increase FRET efficiency and ROS generation^71^. The mixed organic fluorogens gave rise to aggregation-induced emission in a confined area, thus providing bright FL and CL, as well as stable ^1^O_2_ generation. More recently, nanoconjugates consisting of luminol and a transducer (a self-degradable polymer or carbon dot) were reported to be implemented in PDT^72,73^. They also resulted in the successful inhibition of tumor growth with low background cytotoxicity. However, despite the advantages of CL, most studies rely on luminol, which is a high-energy molecule with potential biotoxicity to living systems. In addition, H_2_O_2_ and a catalyst must be added or present in the microenvironment for chemiexcited ^1^O_2_ production. When applied directly in clinical use, these nanoconjugates may cause another toxicity issue and require further optimization.

BL is a subset of CL, in which the light-generating chemical reaction occurs by a process naturally catalyzed by enzymes inside living organisms (e.g., luciferase-luciferin)^74,75^. Therefore, compared to CL, it is anticipated that the photon-emitting capability of BL is more compatible with biological systems. Similar to the advantages observed in proteinaceous PSs, luciferases, as BL-emitting enzymes, can be utilized in the form of either a genetically encoded reporter^76^ or an expressed pure protein^77,78^. Additionally, together with recent advances in detection technology, BL-based assays provide high sensitivity, with levels approaching 10^–20^ moles (equivalent to a few molecules per cell) and a large dynamic range (10^6^ to 10^8^ orders of magnitude). Although BL yields lower light intensities than FL or CL, it is important that a background signal is virtually absent due to the lack of inherent BL reactions in mammalian cells. Therefore, the advantages of BL make it possible to intrinsically regulate ROS production, enabling various PDT approaches. The usability of BL in PDT was initially examined by Theodossiou and coworkers in 2003^79^. They used a genetically encoded firefly luciferase (Fluc) as an intracellular BL source. The coaddition of luciferin (a substrate for Fluc) and rose bengal (a photosensitizing natural dye) into Fluc-transfected cells significantly diminished cell viability due to intracellular ROS production via BL resonance energy transfer (BRET) between the BL compound and rose bengal. Lai’s group^80^ and Yun’s group^81^ demonstrated more advanced BL-based PDTs. The core principle was the conjugation of luciferase protein and semiconducting nanocrystals, quantum dots (QDs). This conjugate led to efficient energy transfer from a BL compound into QDs upon the addition of substrate. Consequently, these enzyme-fused QDs can sensitize various PSs at different wavelengths due to the size-tunable property of QDs. The authors reported that this method effectively suppressed tumor growth by BL, which was not affected by tissue depth. They also used brighter, more stable, and/or redshifted proteins, *Renilla* luciferase (*R*luc) variants and their synthetic substrate coelenterazine. In addition to metallic NPs, it was reported that luciferase was able to be fused with polymeric NPs such as poly(lactic-co-glycolic acid)^82^ or naturally occurring protein NPs such as ferritin^83^ to increase protein stability and/or ROS production in cells, and their abilities to exert PDT were demonstrated in different types of cancer cells.

While these BL-based PDT strategies were successfully employed in conjunction with traditional chemical PSs, they still encountered inherent problems in vivo (e.g., slow degradability or light-sensitive side effects), as mentioned above. To overcome chemical PS toxicity, it was demonstrated that the delivery of genes encoding proteinaceous PS and luciferase was used to kill cancer cells^84^, which would be more beneficial when using tumor-specific promoters. Recently, this idea was further validated using fusion proteins rather than gene expression by Kim’s group^60^. They designed fusion proteins consisting of luciferase, proteinaceous PS, and a tumor-targeting peptide and demonstrated that the protein probe promoted significant cytotoxicity in cancer cells through the generation of BL-sensitive ROS on the plasma membrane, which is denoted by BL-induced proteinaceous PDT (BLiP-PDT). It is important to note that the extremely weak BL intensity (approximately 0.3% lower than that of conventional LED light irradiation) induced a relatively large amount of ROS transiently and locally in proximity to the targeted regions, thus resulting in excellent therapeutic effects in both patient-derived primary cells and tumor-bearing mice. In addition, BLiP-PDT did not encounter light penetration problems in vivo, thus resulting in a therapeutic platform without an external light source.

Based on these findings, the CL- or BL-based approach has great potential in PDT, especially in terms of its simplicity and independence from external light. Nonetheless, these methods still require more research to be clinically useful, which includes improved tumor selectivity and reduced immunogenicity. For example, with a few exceptions^60,70^, most studies relied on passive targeting via EPR, which may adversely affect normal cells, as observed in classical PDTs. Moreover, the long-term in vivo toxicity of some nanomaterials or CL (or BL)-inducing chemical substrates (e.g., luminol, luciferin, or coelenterazine) is of special concern in current studies. In this regard, CL- or BL-based PDTs combined with antibody fragments or nonimmunogenic polymers are required to achieve self-illuminating cancer treatment.

### PDT with oxygen-independent strategies

Tumor hypoxia characterized by deficient tissue oxygenation is a major hurdle in PDT because oxygen is a prerequisite for PDT^85^. Tumor hypoxia can occur in two forms: permanent or transient. In the first form, the rapid progression of the tumor depletes the blood supply, leading to hypoxia^86^. In the second form, PDT itself causes rapid inhibition of the tumor vasculature, leading to rapid depletion of the local oxygen supply^87^. In any case, solid tumors with a high percentage of hypoxic cells have strong resistance to PDT, making it very difficult to continuously perform PDT^88^. The key molecular mechanism of hypoxia begins with the activation of hypoxia-inducible factor-1 (HIF-1), which is a major effector and transcriptional regulator. Not all PDT treatments induce persistent hypoxia, but it was reported that PDT-mediated oxidative stress (especially very long treatments for 12−14 h) led to the activation of HIF-1α^89^. Indeed, this signal transduction pathway provides an important clue to the solution. For example, Heger’s group demonstrated that HIF-1 inhibition not only reduced survival signaling, but also enhanced PDT efficacy, resulting in cancer cell death by PDT even under hypoxic conditions^90,91^.

In addition to the above method, much effort has been made to date to overcome the hypoxia-related problem of PDT. A review article on these strategies was recently published by Yoon’s group^92^. They described three main categories for PDT strategies to avoid hypoxia: i) tumor hypoxia-relieving strategies (e.g., delivery of O_2_ into tumors, regeneration of blood flow, in situ O_2_ generation by decomposing H_2_O_2_, or microvascular alteration), ii) strategies for working with low oxygen concentrations (reduced O_2_ consumption or type I-triggered reaction), and iii) strategies combined PDT with hypoxia-activated therapies (chemotherapy, photothermal therapy (PTT), or immunotherapy). Based on these classifications, we introduce updated research centering on recently published papers without overlap with previous reports.

Han et al.^93^ demonstrated the use of hyperthermia-responsive micelles containing near-infrared (NIR)-responsive PSs, which simultaneously induced thermal cycloreversion for PTT and singlet-oxygen generation for PDT without the participation of tumor oxygen. The small size (~50 nm), positively charged surface (~4 mV), and tumor pH (6.8)-responsive property of the micelles were expected to enhance their cellular binding and tissue penetration, which improved their therapeutic efficiency against multicellular spheroids of 4T1 cancer cells, as well as in a 4T1-xenograft mouse model. This study showed an improved result of oxygen-independent PDT combined with PTT toward hypoxic tumors under mild NIR irradiation. Luo et al. described how hybrid protein oxygen carriers were conjugated with doxorubicin (an anticancer drug) and chlorin e6 (a chemical PS) via the disulfide linkage of hemoglobin and human serum albumin^94^. Owing to its oxygen-carrying ability, the protein hybrid complex supplied oxygen to tumors via caveolae endothelial transcytosis. Concurrently, with the downregulated expression of HIF-1α, multidrug resistance gene 1, and P-glycoprotein, enhanced effects of chemotherapy and PDT were observed. In a more recent study, Zhang and coworkers reported an oxygen self-supplying system using fluorinated photosensitizer-based block copolymers^95^. Owing to the high affinity of fluorine for oxygen, oxygen could be adsorbed into the hydrophobic core in amphiphilic block copolymers and simultaneously delivered to tumor sites. They demonstrated that polymeric NPs were effectively taken up by cancer cells via a pH-responsive route in a weakly acidic tumor microenvironment. Zhao et al. developed a nanocomposite consisting of upconversion NPs (UCNPs) and a NIR light-triggered PS (iridium (III) complex), where an effective HIF-1α inhibitor (YC-1) was physically adsorbed onto the hydrophobic layer at the surfaces of the UCNPs^96^. Since HIF-1α is an attractive target for tumor therapy, and an iridium (III) complex with a long-lived triplet excited state can provide high singlet-oxygen generation, this nanocomposite showed antitumor effects on MDA-MB-231 tumor-bearing mice even at low oxygen content, and permitted deep tissue light penetration.

Given the impact of hypoxia on resistance to tumor therapy, a specific oxygen supply or in situ oxygenation only around the tumor would be the best strategy to achieve the full potential of PDT in a hypoxic tumor. However, when a variety of oxygen generators, oxygen carriers, or nanoconjugates are employed in PDT, the oxygen toxicity in vivo should be carefully considered in future studies, as locally increased partial pressures of oxygen can lead to hyperoxia, which may be detrimental to the lungs and the central nervous system. Furthermore, type II reactions are considered the most common pathway in photodynamic reactions, but it should be mentioned that type III (triplet-doublet interaction) and type IV (photoisomerization) reactions can exert cytotoxic effects directly on intracellular structures without the mediation of oxygen^97^. Therefore, in addition to fundamental research on hypoxia, it is anticipated that the use of novel PS transporters or light-inducible self-regulating PS molecules will be an excellent alternative for the treatment of hypoxic tumors.

## Concluding remarks

**Fig. 2:**
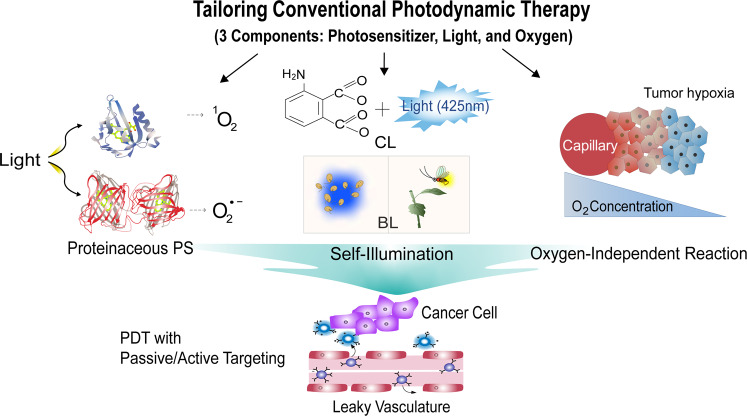
Schematic overview of cancer treatment using smart PDT strategies to overcome the innate problems of conventional PDT. Three PDT approaches, including proteinaceous PS, self-illumination, and oxygen-independent methods are highlighted in this review. CL chemiluminescence, BL bioluminescence.

We discussed various strategies that have improved traditional PDT, focusing on three components of PDT (PS, light, and oxygen), as summarized in Fig. 2. To demonstrate the usefulness of PDT, which has been developed over its long history, extensive research on PDT should be conducted, including studies of safety, biodegradability, and tumor specificity by active targeting, along with examining targeting mechanisms, physicochemical factors, and the physiological characteristics of the tumor microenvironment. While the benchtop-to-bedside translational approach for recently advanced PDTs is still a daunting process, these new insights brighten the clinical outlook for using PDT, thus ensuring next generation strategies. Although not covered in detail in this review, these PDTs can be combined with immunotherapy via immunomodulation, as recently reported elsewhere^98–100^. Therefore, more progressive approaches in conjunction with existing methods or new technologies will promote PDT development for targeted cancer treatment.

In conclusion, we highlighted recent advances in PDT for effective tumor treatment. The key aspect in PDT is ROS generation by light-activated PSs, which leads to tumor and vasculature killing via different cell death pathways and/or immune response activation. However, despite its clinical potential, it is not easy to optimize the PS, light, and oxygen depletion factors of PDT in cancer therapy, primarily due to the complexity of the tumor microenvironment. Beyond the barriers of conventional PDTs, smart approaches in combination with novel bio/nanomaterials or insights have been recently developed, which include the use of proteinaceous PSs, self-illuminating methods, and oxygen-independent strategies. These developed PDTs are necessary to ensure effective and selective delivery to tumor sites as well as in vivo safety, and they have contributed to the increasing usefulness of PDT in cancer treatment. In combination with new materials or other therapeutic modalities, it is anticipated that safer, more effective, and more biocompatible PDT will bring even greater advances in cancer therapy and molecular medicine.
